# Editorial: Bacterial Effectors as Drivers of Human Disease: Models, Methods, Mechanisms

**DOI:** 10.3389/fcimb.2021.708228

**Published:** 2021-07-08

**Authors:** Gunnar N. Schroeder, Jaclyn S. Pearson, Teresa L. M. Thurston

**Affiliations:** ^1^ Wellcome-Wolfson Institute for Experimental Medicine, Queen’s University Belfast, Belfast, United Kingdom; ^2^ Centre for Innate Immunity and Infectious Diseases, Hudson Institute of Medical Research, Clayton, Melbourne, VIC, Australia; ^3^ MRC Centre for Molecular Bacteriology and Infection, Imperial College London, London, United Kingdom

**Keywords:** effectors, bacterial pathogenesis, virulence factors, host-pathogen interactions, secretion systems

## Introduction

Bacteria colonise virtually all ecological niches on earth. To interact with their surroundings, they evolved sophisticated multi-protein secretion systems that transport proteins, called effectors, from the bacterial cytoplasm into the extracellular space or directly into target bacteria or eukaryotic cells. Secretion systems therefore perform critical roles in inter-bacterial competition and the pathogenesis of many clinically important opportunistic and professional human pathogens. Generically numbered type I, type II, etc. secretion systems (T1SSs, T2SSs, …), the complex multi-protein architecture of each type is remarkably conserved in different bacteria (Costa et al., 2015). In contrast, the effector repertoires are diverse in numbers and composition reflecting adaptation to specific lifestyles. Environmental, broad host range pathogens like *Legionella pneumophila* encode more than 300 effectors (Qiu and Luo, 2017). These effectors, whilst not selected under evolutionary pressure by the human immune system, still allow *L. pneumophila* to cause opportunistic infections. In contrast, pathogens with a more restricted mammalian host range, such as *Salmonella enterica*, have a considerably smaller (~20-40) arsenal of effectors (Jennings et al., 2017).

This Frontiers Research Topic comprises a series of reviews and original research articles highlighting common and pathogen-specific findings and approaches to dissect the function of effectors in bacterial pathogenesis.

## The Functional Characterisation of Bacterial Effectors – State of Affairs

Over the last three decades substantial progress has been made in elucidating secretion system structures and secretion mechanisms (Costa et al., 2015; Tran et al., 2021). Functions for a large number of effectors were uncovered, revealing unprecedented activities and host targets; however, many remain uncharacterised and new effectors are still discovered. In a review article Mak and Thurston provide a detailed overview of our current knowledge about the wide range of effector targets and activities, including molecular mimicry of host proteins and completely new protein architectures and enzymatic mechanisms. They also discuss pitfalls and challenges that need to be overcome to further advance the field.

## T3SS Effectors – Injected Munition for Host Subversion by Enteric Pathogens and Beyond

A wide variety of human and environmental pathogens employ T3SSs to translocate effectors in one step from the bacteria into the host cytoplasm (Galan et al., 2014). Many concepts and methods were pioneered during the functional analysis of T3SS effectors and are exemplary and applicable for the investigation of effectors delivered by other secretion systems. Slater and Frankel summarise this knowledge and focus on the *in vivo* approaches available to characterise effectors of enteropathogenic and enterohaemorrhagic *Escherichia coli* and the surrogate mouse pathogen *Citrobacter rodentium*.

The original research paper by Gan et al., is a fine example for the use of several of these assays and state-of the art proteomics, which resulted in the identification of host Rab GTPases as new, physiologically relevant, targets that are glycosylated by the *Salmonella* T3SS effector SseK3.

Understanding the specific roles of effectors in pathogenesis requires not only target identification and biochemical characterisation, but also consideration of how and when the secretion system and effectors are expressed. In a comprehensive review, Kamanova describes the complex framework, in which the T3SS and associated effectors of *Bordetella pertussis* and other classical *Bordetella* species operate to shape host-pathogen interactions. This review also highlights the power of comparative genomics within and beyond species to link genotypes to virulence profiles.

## T4SS Effectors – Overwhelming Numbers Take Control of the Host Cell

Virulence-associated T4SSs are a large group related to conjugation systems. Most prominent is the Dot/Icm T4B subtype found in *L. pneumophila, Coxiella burnetii* and other Legionellales (Duron et al., 2018). Each *L. pneumophila* and *C. burnetii* strain uses its T4BSS to deliver effectors in their hundreds (Qiu and Luo, 2017). The effector repertoires are highly diverse and estimated to comprise more than 18,000 effectors across the *Legionella* genus (Gomez-Valero et al., 2019). Many of these effectors drive the creation of very different replication vacuoles by the two intracellular pathogens (Qiu and Luo, 2017). In their review, Thomas et al. contrast how effectors that exert opposing effects on autophagy, stimulation by *Coxiella* and inhibition by *Legionella*, contribute to this. An original research article by Pechstein et al. illustrates that additional aspects still remain to be discovered. They reveal that the *C. burnetii* effector AnkF is required for efficient intracellular replication and, whilst unable to pin down the exact mechanism, propose that AnkF recruits the intermediate filament vimentin to help stabilise the replication vacuole.

The functions of most *Legionella* effectors still need to be unveiled, but it is clear that *L. pneumophila* dynamically modulates protein ubiquitination (Price and Abu Kwaik, 2021). Some effectors mimic canonical ubiquitin ligases whereas others encode enzymes that mediate ubiquitination *via* completely new chemistry. Further, as highlighted in a review by Kitao et al., *Legionella* also manipulates host ubiquitination *via* effector deubiquitinases that reverse canonical and non-canonical ubiquitination.

## T6SS Effectors – Versatile Weapons for Combating Competitors and Manipulating the Host

Since their discovery in the early 2000s T6SSs have been recognised as one of the most abundant classes of secretion systems in pathogenic and non-pathogenic Gram-negative bacteria and a key role in inter-bacterial competition has been established (Jana and Salomon, 2019). However, important pathogens such as *Vibrio cholerae* use T6SSs to target both bacterial and eukaryotic cells. Here, Monjarás Feria and Valvano review the current knowledge about T6SS effectors that are dedicated to subverting eukaryotic cell signalling, directly promoting virulence.

Moreover, an indirect contribution of antibacterial T6SSs in pathogenesis is emerging. The microbiota is critical for human nutrition and well-being, but also confers colonisation resistance against pathogens through immune priming, nutrient sequestration and physical exclusion (Buffie and Pamer, 2013). Many members of the microbiota express T6SSs. Wood et al. summarise the evidence and discuss how T6SSs in commensals could shape the microbial community and suppress pathogens and how pathogens could employ antibacterial T6SSs to overcome colonisation resistance and disturb microbiota homeostasis, promoting pathogenesis and disease.

The co-existence of anti-bacterial and anti-eukaryotic T6SS effectors in the same isolate raises the interesting question as to how the correct effector is transported in the right moment. T6SSs are phage-derived puncturing devices, in which effectors are loaded onto or fused with the VgrG or PAAR proteins that constitute the tip of the T6SS spike (Jana and Salomon, 2019). Research presented by Wettstadt et al. shows that loading of an *A. tumefaciens* effector on an engineered *P. aeruginosa* VgrG was not sufficient for delivery by the *P. aeruginosa* T6SS, suggesting that additional specificity determinants govern T6SS effector transport.

## T7SS Effectors – Key Virulence Factors of *M. tuberculosis* and Gram-Positive Pathogens

Simultaneously with the increase in understanding of the roles of T6SSs in the biology of Gram-negative bacteria, T7SSs have emerged as central players in the virulence and inter-bacterial interaction of Actinobacteria and other Gram-positives including *Staphylococcus aureus* (Tran et al., 2021). Augenstreich and Briken review the role of the *Mycobacterium tuberculosis* ESX T7SSs, prototypes of this class, and their effectors in the context of other secreted proteins and lipids in the pathogenesis of *Mycobacterium tuberculosis*, the most important bacterial human pathogen.

## Beyond the T7SS: New Systems – New Effectors – New Virulence Mechanisms

New secretion systems and effectors are still discovered. T9SSs form a barrel pore in the outer membrane of *Bacteroidetes* species for surface delivery and release of proteins (Lasica et al., 2017). In a research article, Mu et al. suggest that T9SS-mediated secretion of gingipain proteases by intracellular *Porphyromonas gingivalis* promotes proliferation of colorectal cancer cells *via* MAPK/ERK signalling pathways, adding to an emerging picture of T9SSs as important virulence factors.

A secreted protease is also an important virulence factor of *Campylobacter jejuni*. HtrA, secreted by an unknown pathway, cleaves E-cadherin (Boehm et al., 2018) and, as here revealed by Sharafutdinov et al., the human tight junction protein claudin-8, promoting the opening of cell-to-cell junctions and transmigration of bacteria across the intestinal epithelium.

## Effectors - Inhibitors and Triggers of Immune Signalling

Primary target of many effectors are immune defences, creating an environment conducive to bacterial replication. Most of the current literature has focussed on the manipulation of “classical” antibacterial immune mechanisms such as phagocytosis and pro-inflammatory NF-κB signalling. In a comprehensive review, Alphonse et al. discuss the increasing evidence that type I and type III interferon signalling, well established mediators of antiviral immunity, are also important during bacterial infections and modulated by effectors.

Effectors are undoubtedly powerful tools that enable bacteria to subvert host responses to bacterial danger signals such as peptidoglycan. However, it is becoming increasingly apparent that the presence and activity of effectors do not go unnoticed by mammalian cells, which activate specific effector-triggered immunity (ETI) pathways (Lopes Fischer et al., 2020). Ngwaga et al. review the latest findings on ETI by *Legionella* and discuss the potential of exploiting ETI to combat bacterial infection.

## Conclusions

The articles in this Research Topic illustrate the breadth and celebrate the research that has transformed our understanding of bacterial secretion systems and effector mechanisms over the past ~30 years. They also clearly show that effector biology remains a dynamic field and many exciting discoveries of how effectors manipulate the host, but also how humans sense infection and retaliate lie ahead.

## Author Contributions

All authors made a substantial, direct and intellectual contribution to the work, and approved it for publication.

## Funding

GNS is supported by Medical Research Council UK grant MR/R010552/1. JSP is supported by an Australian National Health and Medical Research Council (NHMRC) Career Development Fellowship (APP1159230). TLMT is supported by a BBSRC David Phillips Fellowship (BB/R011834/1).

## Conflict of Interest

The authors declare that the research was conducted in the absence of any commercial or financial relationships that could be construed as a potential conflict of interest.
